# Supplementary material for: Targeting HSF1 as a Therapeutic Strategy for Multiple Mechanisms of EGFR Inhibitor Resistance in EGFR Mutant Non-Small-Cell Lung Cancer
Source: Cancers (Basel). 2021 Jun 15;13(12):2987. doi: 10.3390/cancers13122987 (PMC8232331; doi:10.3390/cancers13122987)
Supplement: Supplementary file 1 [file cancers-13-02987-s001.zip › cancers-1253451-supplementary/cancers-1253451-original image.pdf]

Sangah Lee, et al

Figure 1C

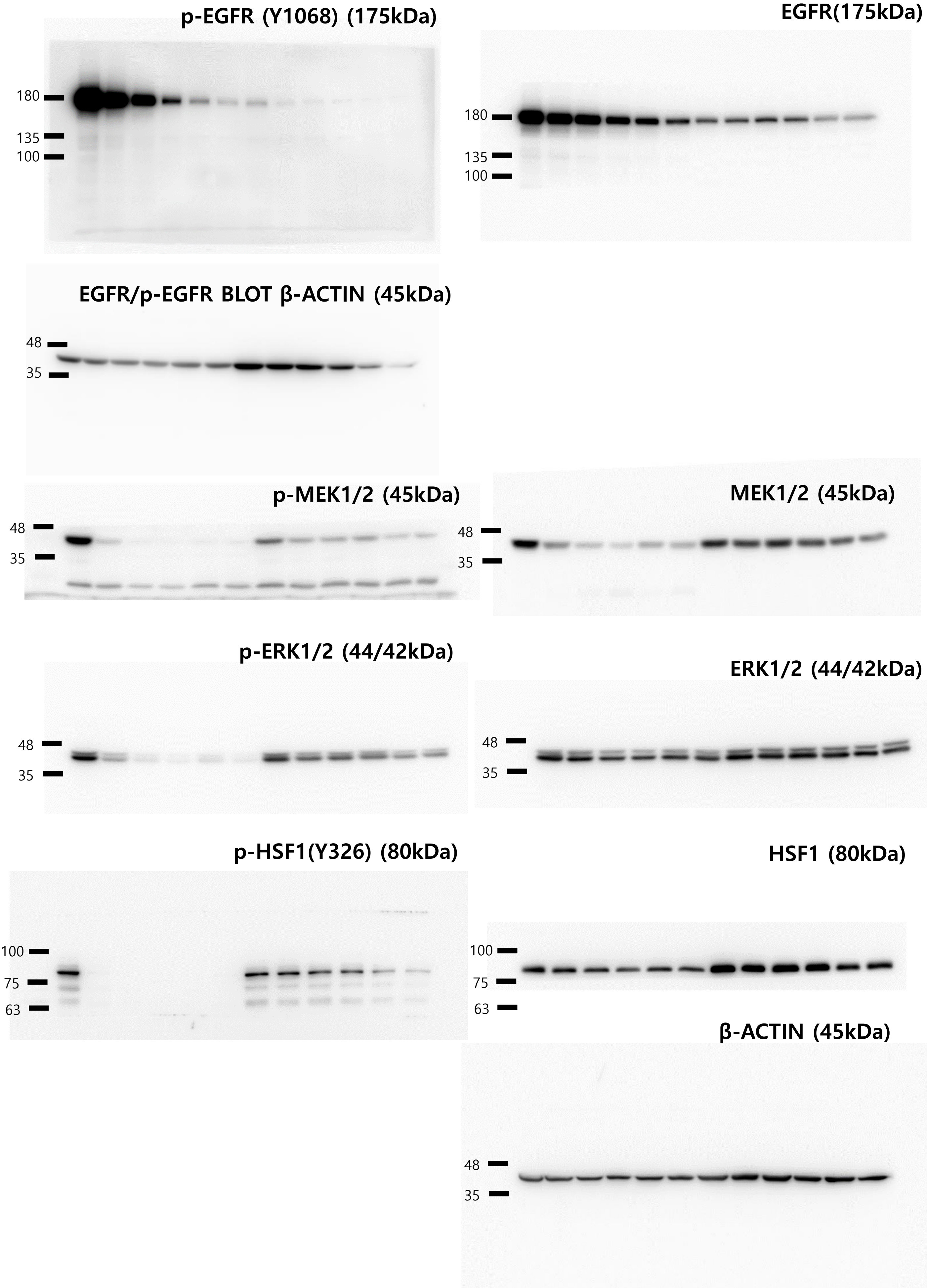

Figure 1C

Name	p-EGFR	EGFR	ACTIN	p-EGF/EGF	EGFR/ACT
HCC827_0μM	51646.35	43563.41	22378.33	1.000000	1.000000
HCC827_0.001μM	34802.14	38258.72	20721.3	0.767287	0.94846
HCC827_0.002μM	23587.83	34921.92	17926.15	0.569734	1.00073
HCC827_0.005μM	8459.75	25055.81	15717.83	0.284794	0.818883
HCC827_0.015μM	3599.92	19909.96	19832.9	0.152512	0.515691
HCC827_0.045μM	2054.89	11980.01	21797.46	0.144682	0.28233
HCC827-ErlR_0μM	2297.48	5972.41	44082.11	1.000000	1.000000
HCC827-ErlR_0.045μM	1009.75	6482.95	41323.02	0.404892	1.15796
HCC827-ErlR_0.1μM	610.48	7857.05	38758.38	0.201981	1.496259
HCC827-ErlR_1μM	152.57	6340.71	28891.6	0.06255	1.619866
HCC827-ErlR_3μM	0.45	903.36	12757.75	0.001295	0.522636
HCC827-ErlR_10μM	30.06	548.07	4737.79	0.142577	0.853833

Name	p-MEK1/2	MEK1/2	ACTIN	p-MKE/MEK	MEK/ACT
HCC827_0μM	37506.85	31437.09	20078.71	1.000000	1.000000
HCC827_0.001μM	6748.76	12968.19	23871.44	0.436191	0.346972
HCC827_0.002μM	1241.56	6565.6	21702.43	0.158498	0.193223
HCC827_0.005μM	588.34	4494.71	23234.14	0.109713	0.123557
HCC827_0.015μM	881.88	8221.53	25423.95	0.089906	0.206539
HCC827_0.045μM	1238.51	8728.5	24365.02	0.11893	0.228805
HCC827-ErlR_0μM	16159.86	26153.27	28453.76	1.000000	1.000000
HCC827-ErlR_0.045μM	8805.97	24225.54	36702.59	0.588291	0.718109
HCC827-ErlR_0.1μM	8078.76	26588.81	39143.69	0.491738	0.739011
HCC827-ErlR_1μM	8633.19	22642.95	35708.77	0.617059	0.689877
HCC827-ErlR_3μM	4535.39	17129.47	37623.64	0.428508	0.495332
HCC827-ErlR_10μM	3931.6	13481.35	31607.62	0.471981	0.46404

Figure 1C

Name	p-ERK1/2	ERK1/2	ACTIN	p-ERK/ERK	ERK/ACT
HCC827_0μM	26436.85	23758.91	20078.71	1.000000	1.000000
HCC827_0.001μM	9526.97	22766.99	23871.44	0.376068	0.806002
HCC827_0.002μM	3006.55	17834.87	21702.43	0.151501	0.694498
HCC827_0.005μM	1489.6	16154.37	23234.14	0.08287	0.587588
HCC827_0.015μM	3190.43	19990.2	25423.95	0.143433	0.664482
HCC827_0.045μM	2258.08	19705.89	24365.02	0.102982	0.6835
HCC827-ErlR_0μM	22603.98	27986.95	28453.76	1.000000	1.000000
HCC827-ErlR_0.045μM	16423.8	25816.61	36702.59	0.787671	0.715133
HCC827-ErlR_0.1μM	16663.67	28620.06	39143.69	0.720893	0.743349
HCC827-ErlR_1μM	15598.22	26452.72	35708.77	0.730088	0.753147
HCC827-ErlR_3μM	9624.54	22957.39	37623.64	0.519073	0.620363
HCC827-ErlR_10μM	9601.7	20847.26	31607.62	0.570256	0.670566

Name	P-HSF1	HSF1	ACTIN	pHSF1/HSF1	HSF1/ACT
HCC827_0μM	15109.91	26354.63	20078.71	1.000000	1.000000
HCC827_0.001μM	910.32	25439.67	23871.44	0.062413	0.811917
HCC827_0.002μM	133.2	22875.99	21702.43	0.010156	0.803064
HCC827_0.005μM	123.99	17802.52	23234.14	0.012148	0.583759
HCC827_0.015μM	129.46	21961.85	25423.95	0.010282	0.65812
HCC827_0.045μM	505.04	22628.51	24365.02	0.038928	0.707568
HCC827-ErlR_0μM	13309.45	41731.48	28453.76	1.000000	1.000000
HCC827-ErlR_0.045μM	11676.78	37794.67	36702.59	0.968715	0.702117
HCC827-ErlR_0.1μM	9564.15	41414.32	39143.69	0.724102	0.721381
HCC827-ErlR_1μM	8420.9	36862.86	35708.77	0.716264	0.703866
HCC827-ErlR_3μM	4070.3	26354.59	37623.64	0.484255	0.477608
HCC827-ErlR_10μM	2262.06	28251.27	31607.62	0.251056	0.609428

Sangah Lee, et al

Figure 1D

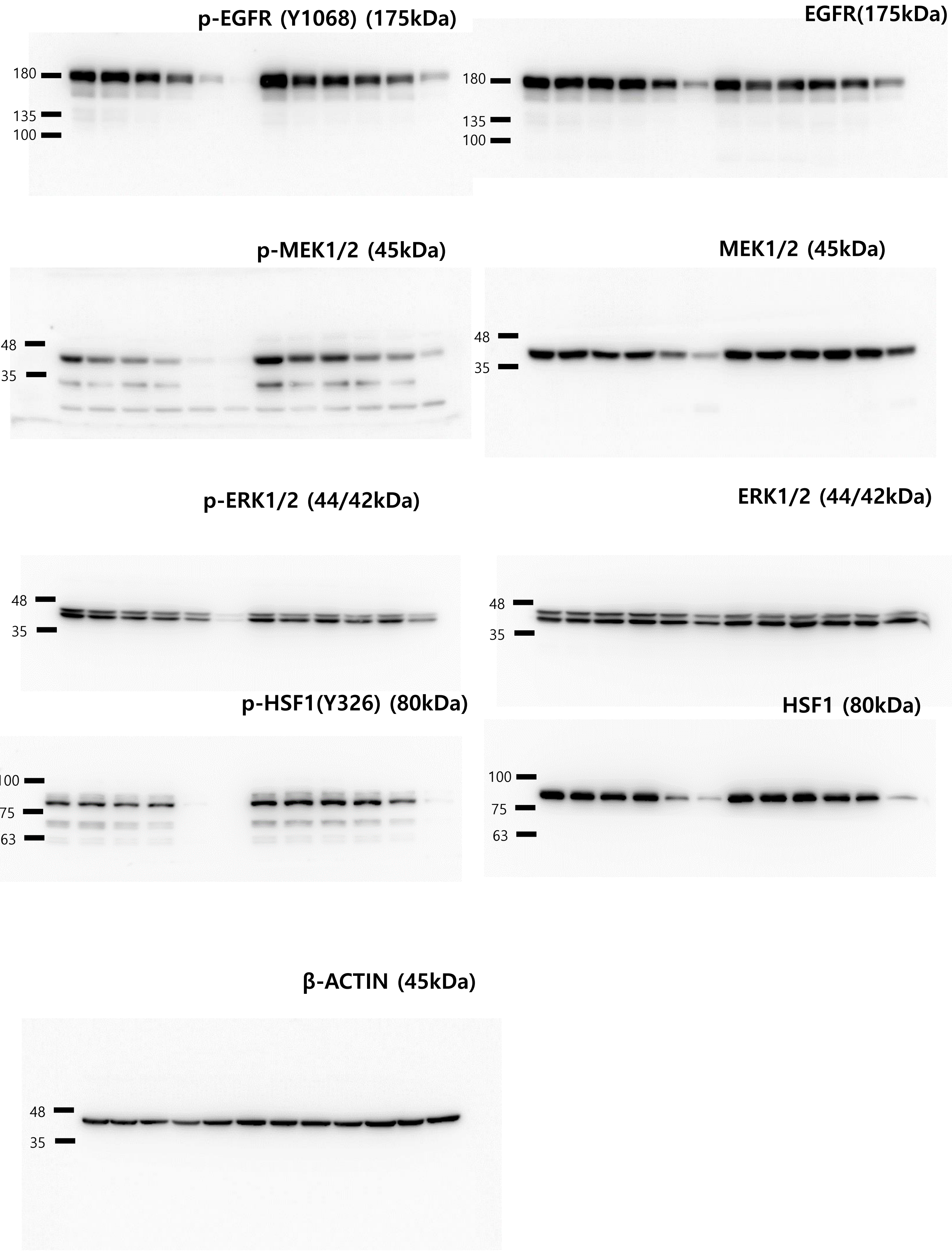

Figure 1D Sangah Lee, et al

Name	p-EGFR	EGFR	ACTIN	p-EGF/EGF	EGFR/ACT
PC9_0μM	42442.12	43255.08	34170.61	1.000000	1.000000
PC9_0.001μM	45533.22	43390.27	34076.51	1.069488	1.005895
PC9_0.002μM	37579.05	42627.15	31982.53	0.898462	1.052905
PC9_0.005μM	26088.47	42679.37	26733.88	0.622975	1.261164
PC9_0.015μM	7971.58	31216.35	37256.67	0.260257	0.661902
PC9_0.045μM	3376.24	12868.44	42861.87	0.267391	0.237176
PC9-ErlR_0μM	53177.84	39945.92	40649.33	1.000000	1.000000
PC9-ErlR_0.045μM	39383.75	29361.27	41197.17	1.007590	0.725251
PC9-ErlR_0.1μM	41133.04	33727.66	38416.52	0.916107	0.893407
PC9-ErlR_1μM	32669.55	33882.84	45776.57	0.724278	0.753213
PC9-ErlR_3μM	25048.84	28403.21	43519.82	0.662463	0.664143
PC9-ErlR_10μM	9117.11	14591.54	45332.93	0.469351	0.327543

Name	p-MEK1/2	MEK1/2	ACTIN	p-MKE/MEK	MEK/ACT
PC9_0μM	25290.34	36876.08	34170.61	1.000000	1.000000
PC9_0.001μM	20236.07	39827.9	34076.51	0.740848	1.083029
PC9_0.002μM	16072.03	33485.49	31982.53	0.699849	0.970179
PC9_0.005μM	9370.14	35155.45	26733.88	0.388636	1.218537
PC9_0.015μM	2845.46	22094.23	37256.67	0.187786	0.549519
PC9_0.045μM	2255.75	11622.33	42861.87	0.283001	0.251264
PC9-ErlR_0μM	41902.58	41843.5	40649.33	1.000000	1.000000
PC9-ErlR_0.045μM	29840.45	41218.78	41197.17	0.722932	0.971971
PC9-ErlR_0.1μM	32104.57	44024.79	38416.52	0.728210	1.113281
PC9-ErlR_1μM	19358.37	45785.29	45776.57	0.422211	0.971646
PC9-ErlR_3μM	15760.69	41150.77	43519.82	0.382459	0.918578
PC9-ErlR_10μM	5963.56	28271.06	45332.93	0.210645	0.605834

Figure 1D Sangah Lee, et al

Name	p-ERK1/2	ERK1/2	ACTIN	p-ERK/ERK	ERK/ACT
PC9_0μM	33308.14	22456.68	34170.61	1.000000	1.000000
PC9_0.001μM	31890.65	27161.06	34076.51	0.791611	1.212827
PC9_0.002μM	26041.68	31329.3	31982.53	0.560420	1.490545
PC9_0.005μM	24096.77	31562.58	26733.88	0.514732	1.796461
PC9_0.015μM	16506.82	28801.53	37256.67	0.386405	1.176302
PC9_0.045μM	4496.58	21962.38	42861.87	0.138038	0.779678
PC9-ErlR_0μM	28324.78	30615.13	40649.33	1.000000	1.000000
PC9-ErlR_0.045μM	24765.48	30903.61	41197.17	0.866178	0.995999
PC9-ErlR_0.1μM	26478.96	32916.68	38416.52	0.869470	1.137667
PC9-ErlR_1μM	21199.28	32090.73	45776.57	0.714021	0.930794
PC9-ErlR_3μM	24202.16	28352.46	43519.82	0.922641	0.865010
PC9-ErlR_10μM	12585.2	27252.47	45332.93	0.499142	0.798196

Name	P-HSF1	HSF1	ACTIN	pHSF1/HSF1	HSF1/ACT
PC9_0μM	20189.61	40086.94	34170.61	1.000000	1.000000
PC9_0.001μM	22259.77	38931.38	34076.51	1.135261	0.973855
PC9_0.002μM	17875.45	37255.36	31982.53	0.952672	0.992946
PC9_0.005μM	18777.04	39609.26	26733.88	0.941251	1.262945
PC9_0.015μM	1271.74	16129.29	37256.67	0.156552	0.369029
PC9_0.045μM	991.22	7928.65	42861.87	0.248225	0.157680
PC9-ErlR_0μM	30073.35	40793.96	40649.33	1.000000	1.000000
PC9-ErlR_0.045μM	32311.63	42073.82	41197.17	1.041744	1.017659
PC9-ErlR_0.1μM	29991.31	40849.51	38416.52	0.995916	1.059562
PC9-ErlR_1μM	28522.93	37646.83	45776.57	1.027732	0.819488
PC9-ErlR_3μM	16750.21	30668.11	43519.82	0.740879	0.702195
PC9-ErlR_10μM	948.16	7504.81	45332.93	0.171378	0.164962

Figure 1E Sangah Lee, et al

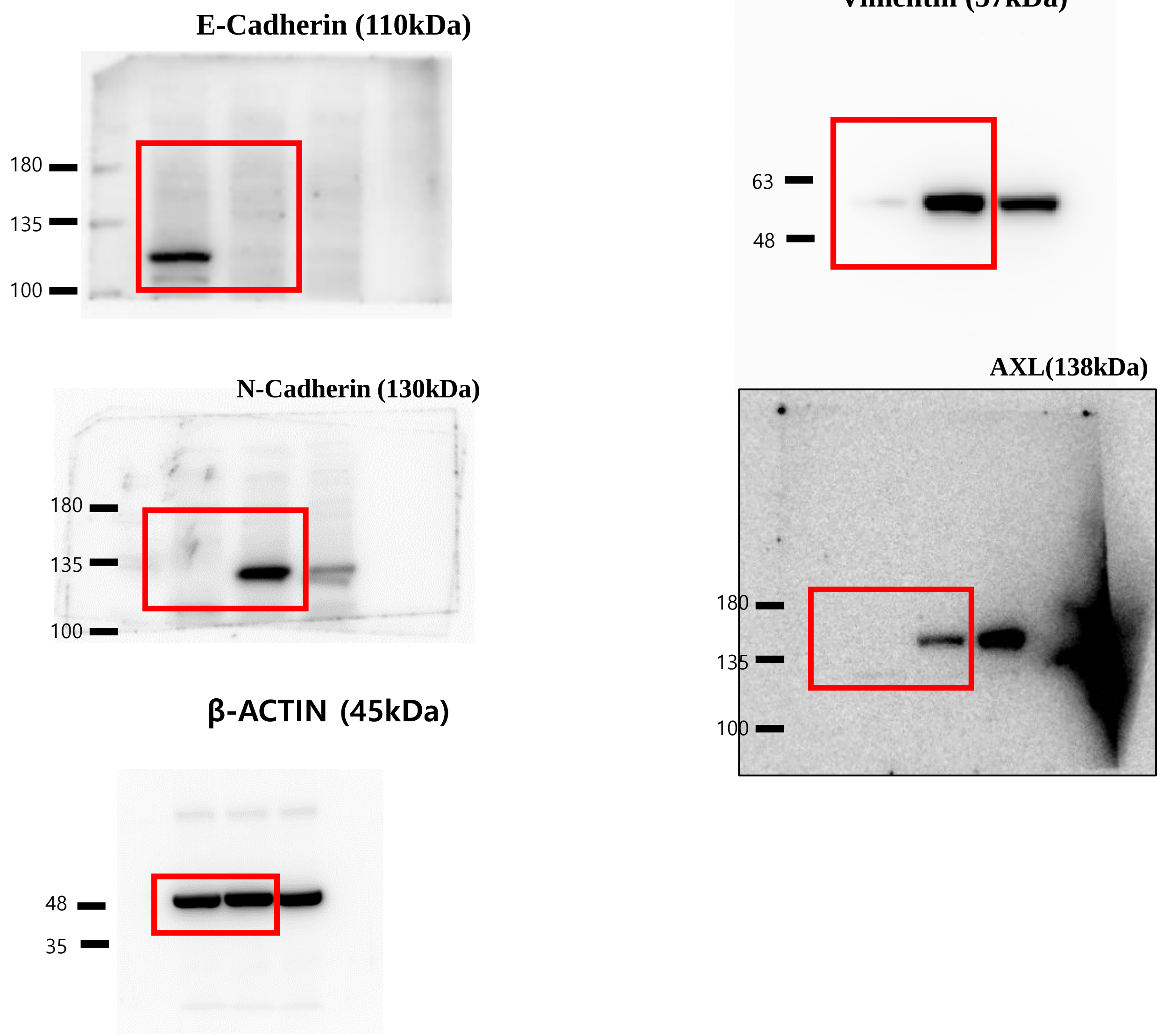

Name	E-cadherin	Vimentin	ACTIN	E-CAD/ACT	VIM/ACT
HCC827_PAR	37267.41	2507.51	43434.03	1.000000	1.000000
HCC827_RES	6620.64	45790.46	48925.46	0.157712	16.211662

Name	N-cadherin	AXL	ACTIN	N-CAD/ACT	AXL/ACT
HCC827_PAR	4758.57	81.07	43434.03	1.000000	1.000000
HCC827_RES	42348.27	3196.32	48925.46	7.900497	38.961210

Figure 3C

Sangah Lee, et al

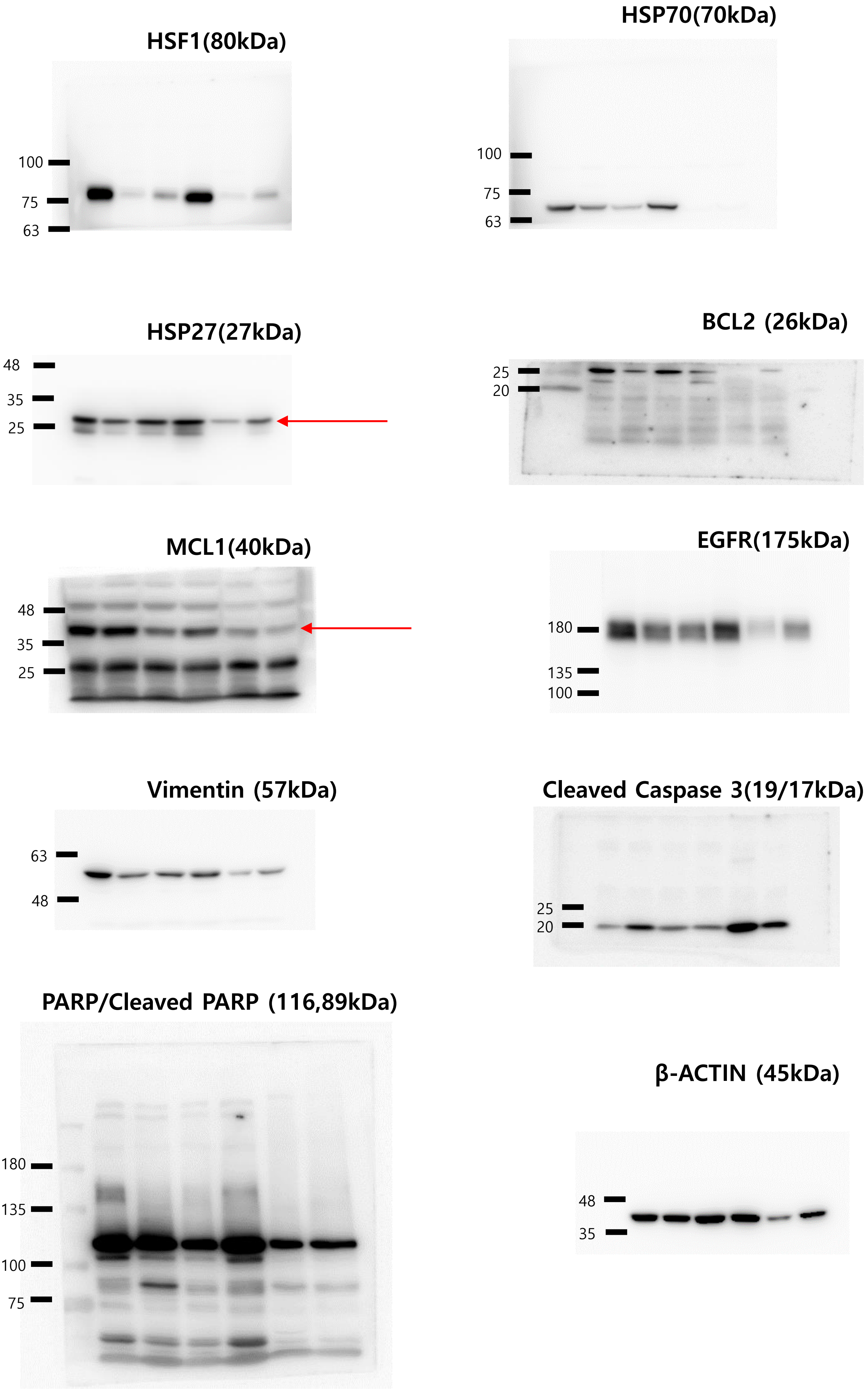

Name	HSF1	HSP70	HSP27	ACTIN	HSF/ACT	H70/ACT	H27/ACT
D3_CTL shRNA	41414.84	20319.41	22229.21	35388.73	1.000000	1.000000	1.000000
D3_HSF1 shRNA_#1	4962.92	12022.68	12684	37752.71	0.112331	0.554635	0.534871
D3_HSF1 shRNA_#2	9113.53	6677.3	22157.34	45210.17	0.172250	0.257228	0.780230
D5_CTL shRNA	36626.81	18318.2	27976.8	43202.15	1.000000	1.000000	1.000000
D5_HSF1 shRNA_#1	2890.82	1033.04	911.28	14258.74	0.239137	0.170867	0.098691
D5_HSF1 shRNA_#2	8589.73	631.75	7428.81	28799.41	0.351805	0.051735	0.398330

Name	BCL2	MCL1	EGFR	ACTIN	BC/ACT	MC/ACT	EGF/ACT
D3_CTL shRNA	20015.12	35247.94	35882.92	35388.73	1.000000	1.000000	1.000000
D3_HSF1 shRNA_#1	6816.73	35995.3	25268.31	37752.71	0.319253	0.957258	0.660093
D3_HSF1 shRNA_#2	16611.21	18662	22889.56	45210.17	0.649639	0.414432	0.499319
D5_CTL shRNA	6120.22	22031.23	32387.79	43202.15	1.000000	1.000000	1.000000
D5_HSF1 shRNA_#1	48.11	12888.21	8114.67	14258.74	0.023817	1.772467	0.759126
D5_HSF1 shRNA_#2	2328.18	8991.09	15752.3	28799.41	0.570652	0.612203	0.729599

Name	VIMEINT	Cleaved Caspase 3	ACTIN	VIM/ACT	CC3/ACT
D3_CTL shRNA	22017.81	5452.38	35388.73	1.000000	1.000000
D3_HSF1 shRNA_#1	10187.06	14246.55	37752.71	0.433702	2.449291
D3_HSF1 shRNA_#2	10529.48	9200.93	45210.17	0.374336	1.320914
D5_CTL shRNA	12590.16	9165.91	43202.15	1.000000	1.000000
D5_HSF1 shRNA_#1	3382.11	32389.11	14258.74	0.813918	10.706503
D5_HSF1 shRNA_#2	5753.13	16298.93	28799.41	0.685480	2.667505

Figure 4C

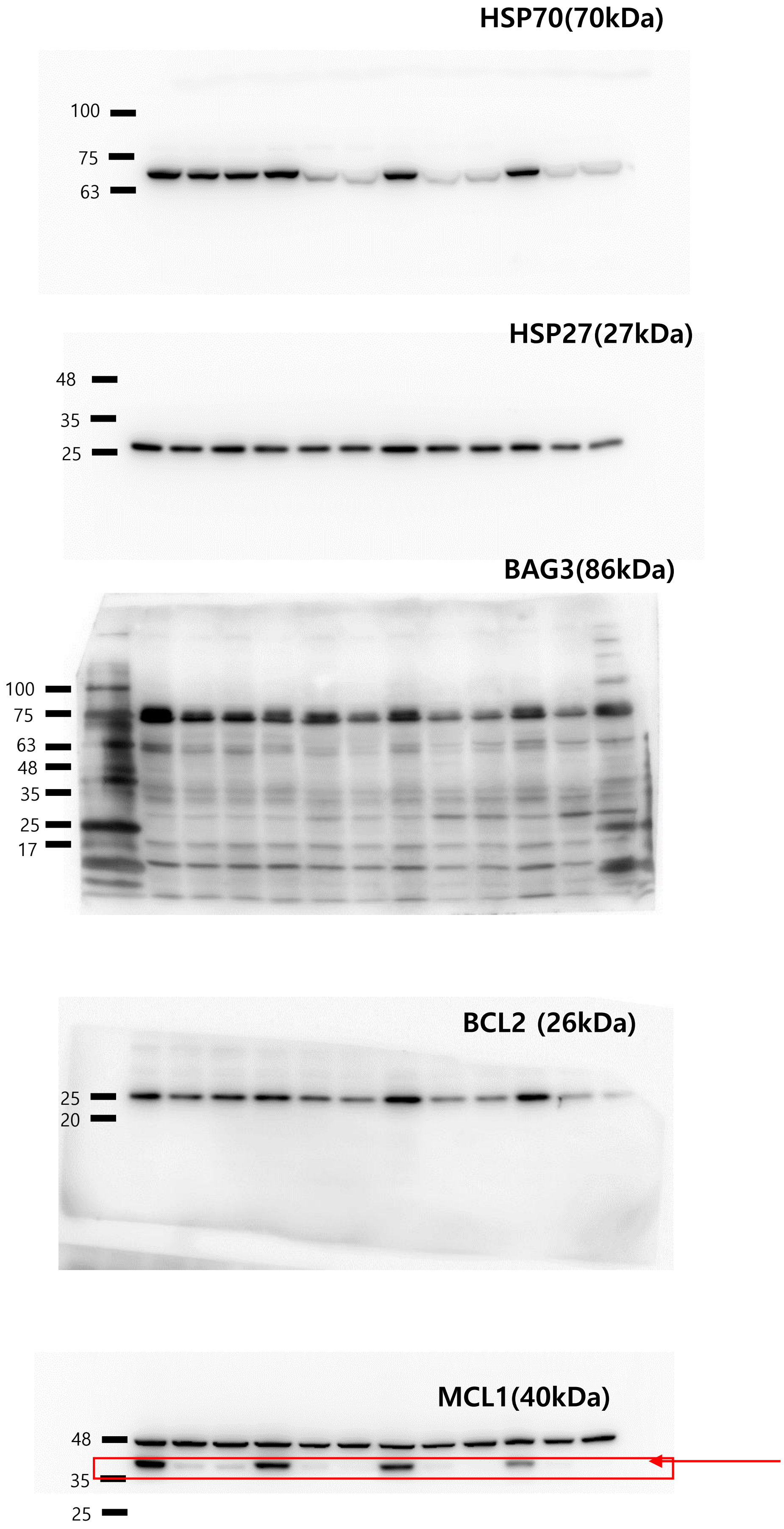
HSP70(70kDa)
100
75
63
HSP27(27kDa)
48
35
25
BAG3(86kDa)
100
75
63
48
35
25
17
BCL2 (26kDa)
25
20
MCL1(40kDa)
48
35
25

Figure 4C

Sangah Lee, et al

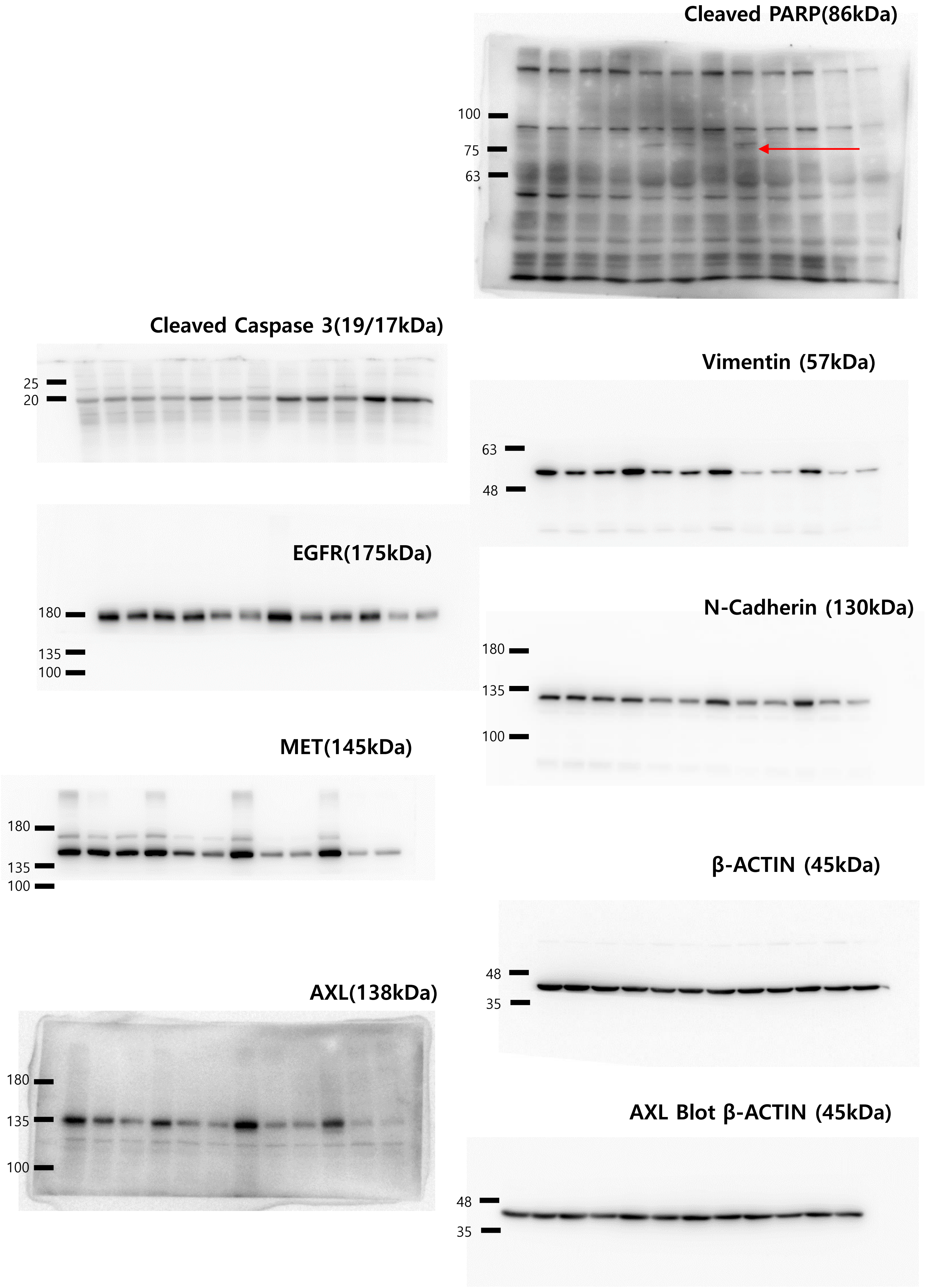

Figure 4C

Name	HSP70	HSP27	ACTIN	HSP70/ACT	HSP27/ACT
827-ErlR_0μM_12hr	38940.3	31373.92	45534.9	1.000000	1.000000
827-ErlR_10μM_12hr	34234.3	28428.02	50411.47	0.794104	0.818451
827-ErlR_20μM_12hr	34721.82	33546.52	43529.08	0.932756	1.118520
827-ErlR_0μM_24hr	38457.12	29225.64	39287.35	1.000000	1.000000
827-ErlR_10μM_24hr	14597.72	26659	32269.97	0.462128	1.110539
827-ErlR_20μM_24hr	10481.53	26471.59	35373.62	0.302706	1.005980
827-ErlR_0μM_48hr	34930.49	36773.55	38848.48	1.000000	1.000000
827-ErlR_10μM_48hr	8178.49	28744.05	41342.91	0.220010	0.734489
827-ErlR_20μM_48hr	10647.04	28198.91	39363.7	0.300817	0.756789
827-ErlR_0μM_72hr	33533.05	28604.01	41511	1.000000	1.000000
827-ErlR_20μM_72hr	10289.88	19762.87	38408.64	0.331644	0.746719
827-ErlR_20μM_72hr	8911.4	18119.1	38052.8	0.289901	0.691013

Name	BCL2	MCL1	ACTIN	BCL/ACT	MCL/ACT
827-ErlR_0μM_12hr	17556.92	36724.36	45534.9	1.000000	1.000000
827-ErlR_10μM_12hr	14105.87	8192.52	50411.47	0.725716	0.201502
827-ErlR_20μM_12hr	17400.63	7286.63	43529.08	1.036768	0.207557
827-ErlR_0μM_24hr	19604.86	33874.09	39287.35	1.000000	1.000000
827-ErlR_10μM_24hr	13284.59	5496.63	32269.97	0.824971	0.197553
827-ErlR_20μM_24hr	11490.46	4366.19	35373.62	0.650949	0.143156
827-ErlR_0μM_48hr	24063.96	28770.77	38848.48	1.000000	1.000000
827-ErlR_10μM_48hr	9564.15	4475.29	41342.91	0.373467	0.146165
827-ErlR_20μM_48hr	9291.56	2808.22	39363.7	0.381066	0.096329
827-ErlR_0μM_72hr	20253.1	15446.5	41511	1.000000	1.000000
827-ErlR_20μM_72hr	7185.8	2470.68	38408.64	0.383458	0.172870
827-ErlR_20μM_72hr	2353.32	964.25	38052.8	0.126755	0.068098

Figure 4C

Name	BAG3	VIMEINT	ACTIN	BAG3/ACT	VIM/ACT
827-ErlR_0μM_12hr	26708.97	28317.44	45534.9	1.000000	1.000000
827-ErlR_10μM_12hr	19504.19	18149.88	50411.47	0.659608	0.578942
827-ErlR_20μM_12hr	18417.33	19190.32	43529.08	0.721331	0.708913
827-ErlR_0μM_24hr	17849.93	34955.18	39287.35	1.000000	1.000000
827-ErlR_10μM_24hr	19504.92	15156.96	32269.97	1.330337	0.527904
827-ErlR_20μM_24hr	12165.86	15063.16	35373.62	0.756971	0.478606
827-ErlR_0μM_48hr	21396.31	27683.75	38848.48	1.000000	1.000000
827-ErlR_10μM_48hr	10172.14	5751.41	41342.91	0.446731	0.195219
827-ErlR_20μM_48hr	9615.64	6230.52	39363.7	0.443524	0.222115
827-ErlR_0μM_72hr	18992.1	18427.55	41511	1.000000	1.000000
827-ErlR_20μM_72hr	8826.78	3991.79	38408.64	0.502300	0.234118
827-ErlR_20μM_72hr	19406.96	4612.3	38052.8	1.114708	0.273040

Name	EGFR	N-cadherin	ACTIN	EGF/ACT	N-CAD/ACT
827-ErlR_0μM_12hr	34641.66	24222.79	45534.9	1.000000	1.000000
827-ErlR_10μM_12hr	29526.75	25192.63	50411.47	0.769896	0.939430
827-ErlR_20μM_12hr	34327.27	23942.8	43529.08	1.036586	1.033988
827-ErlR_0μM_24hr	33693.85	22925.94	39287.35	1.000000	1.000000
827-ErlR_10μM_24hr	22891.15	15607.75	32269.97	0.827125	0.828834
827-ErlR_20μM_24hr	20943.92	13766.24	35373.62	0.690368	0.666901
827-ErlR_0μM_48hr	43962.45	27351.3	38848.48	1.000000	1.000000
827-ErlR_10μM_48hr	23830.48	15727.59	41342.91	0.509359	0.540328
827-ErlR_20μM_48hr	26637.13	13996.92	39363.7	0.597976	0.505048
827-ErlR_0μM_72hr	31114.77	26944.19	41511	1.000000	1.000000
827-ErlR_20μM_72hr	12147.78	12482.08	38408.64	0.421953	0.500675
827-ErlR_20μM_72hr	13867.06	7146.05	38052.8	0.486177	0.289319

Figure 4C

Name	MET	AXL	ACTIN	AXL-ACTIN	MET/ACT	AXL/ACT
827-ErlR_0μM_12hr	44966.14	5301.68	45534.9	19274.39	1.000000	1.000000
827-ErlR_10μM_12hr	43568.89	3697.87	50411.47	22833.14	0.875197	0.588780
827-ErlR_20μM_12hr	40931.75	2257.18	43529.08	24073.95	0.952225	0.340868
827-ErlR_0μM_24hr	45782.93	4124.34	39287.35	19002.14	1.000000	1.000000
827-ErlR_10μM_24hr	28726.8	2252.9	32269.97	26212.06	0.763902	0.395994
827-ErlR_20μM_24hr	23297.78	1544.34	35373.62	22608.81	0.565177	0.314712
827-ErlR_0μM_48hr	47763.44	6586.21	38848.48	27052.67	1.000000	1.000000
827-ErlR_10μM_48hr	18218.14	2017.57	41342.91	25189.8	0.358411	0.328987
827-ErlR_20μM_48hr	16958.03	2206.74	39363.7	25010.9	0.350395	0.362407
827-ErlR_0μM_72hr	45510.97	4291.97	41511	19234.02	1.000000	1.000000
827-ErlR_20μM_72hr	11855.23	1025.62	38408.64	22637.07	0.281532	0.203039
827-ErlR_20μM_72hr	12161.71	599.29	38052.8	18468.53	0.291511	0.145418

Figure 4D

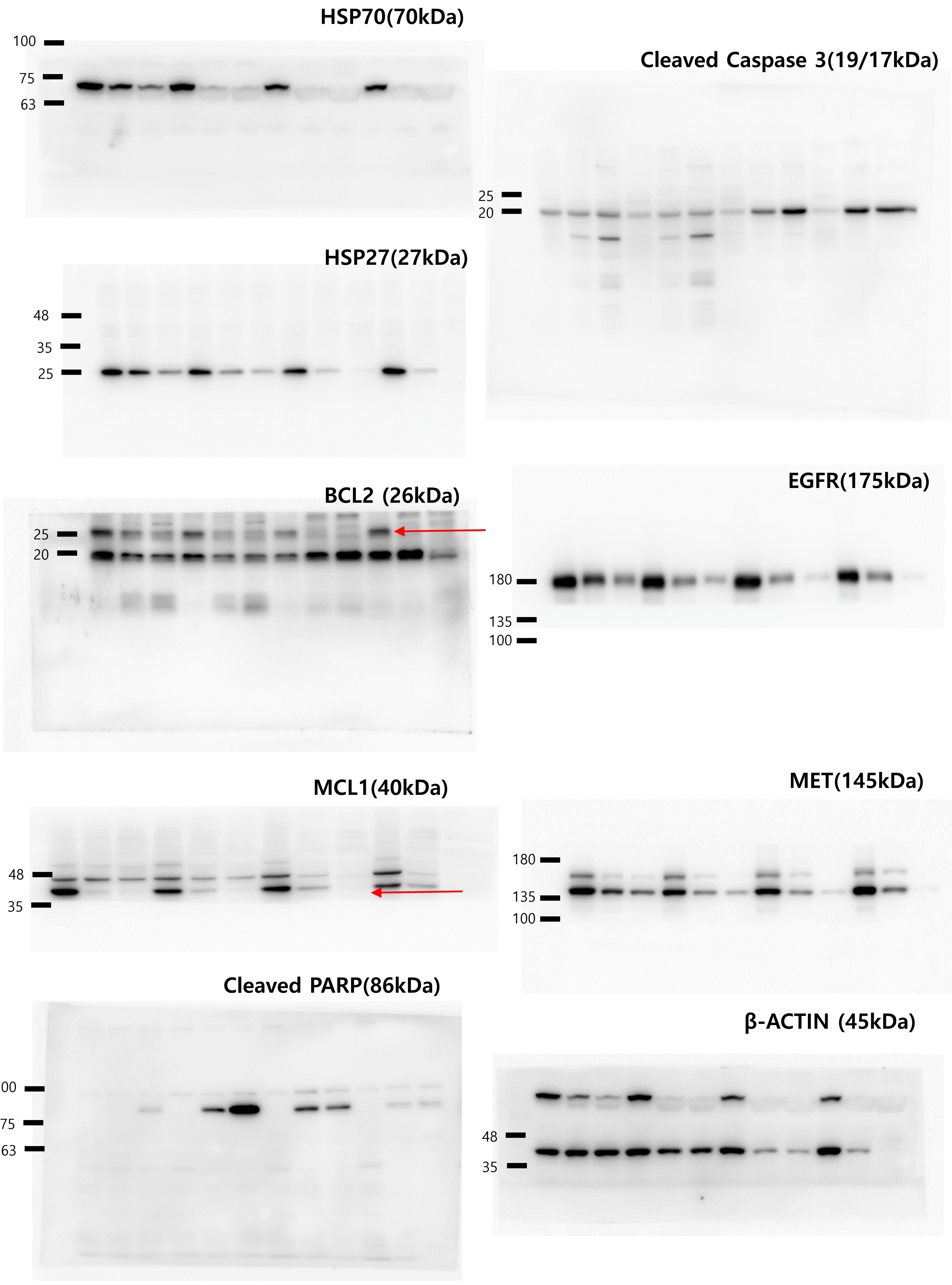
HSP70(70kDa)
100
75
63
Cleaved Caspase 3(19/17kDa)
25
20
HSP27(27kDa)
48
35
25
BCL2 (26kDa)
25
20
EGFR(175kDa)
180
135
100
MCL1(40kDa)
48
35
MET(145kDa)
180
135
100
Cleaved PARP(86kDa)
00
75
63
β-ACTIN (45kDa)
48
35

Figure 4D

Name	HSP70	HSP27	ACTIN	HSP70/ACT	HSP27/ACT
827_0μM_12hr	41552.92	30291.51	37626.64	1.000000	1.000000
827_10μM_12hr	29779.26	21608.92	39481.72	0.682986	0.679848
827_20μM_12hr	18263.19	10610.82	41054.47	0.402819	0.321043
827_0μM_24hr	39626.02	26305.12	43210.31	1.000000	1.000000
827_10μM_24hr	9696.65	9739.83	32100.38	0.329396	0.498412
827_20μM_24hr	8109.28	5721.09	30674.05	0.288282	0.306376
827_0μM_48hr	29162.72	23558.68	40260.94	1.000000	1.000000
827_10μM_48hr	5061.19	4316.92	12395.57	0.563692	0.595169
827_20μM_48hr	3801.55	1064.69	8867.88	0.591830	0.205181
827_0μM_72hr	27000.06	32714.78	43198.74	1.000000	1.000000
827_20μM_72hr	5366.45	3601	10410.63	0.824739	0.456744
827_20μM_72hr	3130.3	5.2	309.76	16.168397	0.022167

Name	BCL2	MCL1	ACTIN	BCL/ACT	MCL/ACT
827_0μM_12hr	28427.16	42895.03	37626.64	1.000000	1.000000
827_10μM_12hr	13797.35	9444.83	39481.72	0.462553	0.209839
827_20μM_12hr	9380.18	6378.63	41054.47	0.302422	0.136287
827_0μM_24hr	19158.8	32951.74	43210.31	1.000000	1.000000
827_10μM_24hr	6520.32	9507.2	32100.38	0.458118	0.388375
827_20μM_24hr	7347.38	5095.91	30674.05	0.540232	0.217851
827_0μM_48hr	11002.21	37651.88	40260.94	1.000000	1.000000
827_10μM_48hr	5863.68	12253.95	12395.57	1.731043	1.057078
827_20μM_48hr	5503.7	4228.09	8867.88	2.271114	0.509826
827_0μM_72hr	22360.33	26535.17	43198.74	1.000000	1.000000
827_20μM_72hr	4420.86	12005.47	10410.63	0.820394	1.877376
827_20μM_72hr	2296.9	3105	309.76	14.325495	16.318697

Figure 4D

Name	EGFR	MET	ACTIN	EGF/ACT	MET/ACT
827_0μM_12hr	46132.4	43704.52	37626.64	1.000000	1.000000
827_10μM_12hr	32958.21	26000.79	39481.72	0.680859	0.566969
827_20μM_12hr	20003.42	19319.52	41054.47	0.397405	0.405140
827_0μM_24hr	44947.06	34847.33	43210.31	1.000000	1.000000
827_10μM_24hr	20084.98	14032.27	32100.38	0.601516	0.542045
827_20μM_24hr	11426	9383.19	30674.05	0.358104	0.379313
827_0μM_48hr	47150.3	37450.58	40260.94	1.000000	1.000000
827_10μM_48hr	16917.99	13658.44	12395.57	1.165418	1.184568
827_20μM_48hr	4694.65	5006.66	8867.88	0.452046	0.606951
827_0μM_72hr	37736.56	42631.95	43198.74	1.000000	1.000000
827_20μM_72hr	18646.44	19732.04	10410.63	2.050349	1.920573
827_20μM_72hr	2024.86	1646.12	309.76	7.483046	5.384831

Figure 5B

Sangah Lee, et al

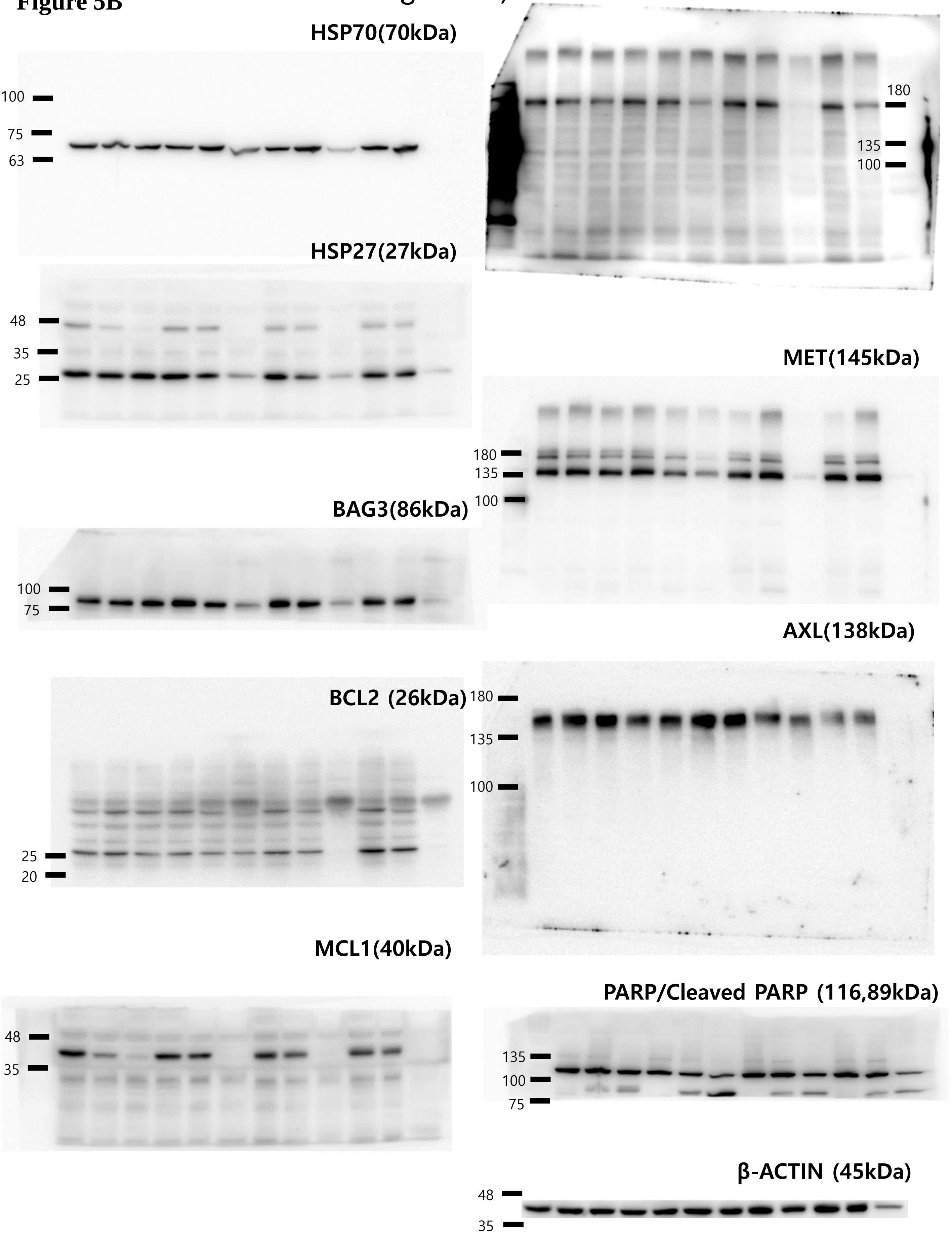

Figure 5B

Name	HSP70	HSP27	ACTIN	HSP70/ACT	HSP27/ACT
H820_0μM_12hr	104.41	25167.75	26843.71	1.000000	1.000000
H820_10μM_12hr	100.36	20761.28	31399.57	0.821746	0.705226
H820_20μM_12hr	107.82	22656.28	30262.67	0.915994	0.798508
H820_0μM_24hr	106.72	29512.04	29599.52	1.000000	1.000000
H820_10μM_24hr	123.25	22453.14	31277.83	1.092922	0.719989
H820_20μM_24hr	84.45	17007.45	35481.74	0.660136	0.480750
H820_0μM_48hr	105.69	12476.68	31284.77	1.000000	1.000000
H820_10μM_48hr	129.04	10810.9	35154.73	1.086525	0.771102
H820_20μM_48hr	38.14	9425.89	28656.85	0.393959	0.824760
H820_0μM_72hr	117.32	22355.51	36171.6	1.000000	1.000000
H820_20μM_72hr	127.11	21523.8	30876.48	1.269251	1.127910
H820_20μM_72hr	2.24	20440.26	12599.73	0.054813	2.624873

Name	BCL2	MCL1	ACTIN	BCL/ACT	MCL/ACT
H820_0μM_12hr	9012.13	38353.3	26843.71	1.000000	1.000000
H820_10μM_12hr	9282.51	16633.28	31399.57	0.880556	0.370761
H820_20μM_12hr	5999.04	8394.05	30262.67	0.590459	0.194135
H820_0μM_24hr	6901.51	33807.33	29599.52	1.000000	1.000000
H820_10μM_24hr	4666.84	27547.53	31277.83	0.639922	0.771116
H820_20μM_24hr	4065	2932.14	35481.74	0.491356	0.072353
H820_0μM_48hr	8522.6	29908.54	31284.77	1.000000	1.000000
H820_10μM_48hr	6707.86	22835.94	35154.73	0.700424	0.679474
H820_20μM_48hr	391.9	2543.54	28656.85	0.050200	0.092843
H820_0μM_72hr	13607.57	28481.96	36171.6	1.000000	1.000000
H820_20μM_72hr	12597.66	20098.59	30876.48	1.084549	0.826677
H820_20μM_72hr	316.1	700.48	12599.73	0.066688	0.070604

Figure 5B

Name	BAG3	EGFR	ACTIN	BAG3/ACT	EGFR/ACT
H820_0µM_12hr	25700.42	11572.07	26843.71	1.000000	1.000000
H820_10µM_12hr	28745.74	10799.75	31399.57	0.956207	0.797850
H820_20µM_12hr	31589.21	7277.6	30262.67	1.090269	0.557844
H820_0µM_24hr	37471.53	11349.03	29599.52	1.000000	1.000000
H820_10µM_24hr	27038.16	10914.74	31277.83	0.682848	0.910128
H820_20µM_24hr	12450.87	5100.04	35481.74	0.277190	0.374882
H820_0µM_48hr	32546.67	12579.9	31284.77	1.000000	1.000000
H820_10µM_48hr	29285.43	13005.39	35154.73	0.800745	0.920016
H820_20µM_48hr	7992.55	936.34	28656.85	0.268092	0.081257
H820_0µM_72hr	27691.86	15813.36	36171.6	1.000000	1.000000
H820_20µM_72hr	25533.64	10564.9	30876.48	1.080191	0.782674
H820_20µM_72hr	3098.75	270.46	12599.73	0.321248	0.049100

Name	MET	AXL	ACTIN	MET/ACT	AXL/ACT
H820_0µM_12hr	32836.66	9733.41	26843.71	1.000000	1.000000
H820_10µM_12hr	34903.89	13073.09	31399.57	0.908728	1.148238
H820_20µM_12hr	30699.82	15083	30262.67	0.829301	1.374542
H820_0µM_24hr	34937.56	10259.69	29599.52	1.000000	1.000000
H820_10µM_24hr	22693.12	11022.59	31277.83	0.614681	1.016711
H820_20µM_24hr	15431.14	16785.63	35481.74	0.368456	1.364844
H820_0µM_48hr	30602.41	15458.71	31284.77	1.000000	1.000000
H820_10µM_48hr	38203.28	9980.51	35154.73	1.110949	0.574551
H820_20µM_48hr	4616.5	8043.94	28656.85	0.164688	0.568068
H820_0µM_72hr	35718.34	6211.07	36171.6	1.000000	1.000000
H820_20µM_72hr	41277.77	7511.94	30876.48	1.353832	1.416856
H820_20µM_72hr	1612.18	592	12599.73	0.129577	0.273629

Figure 5D

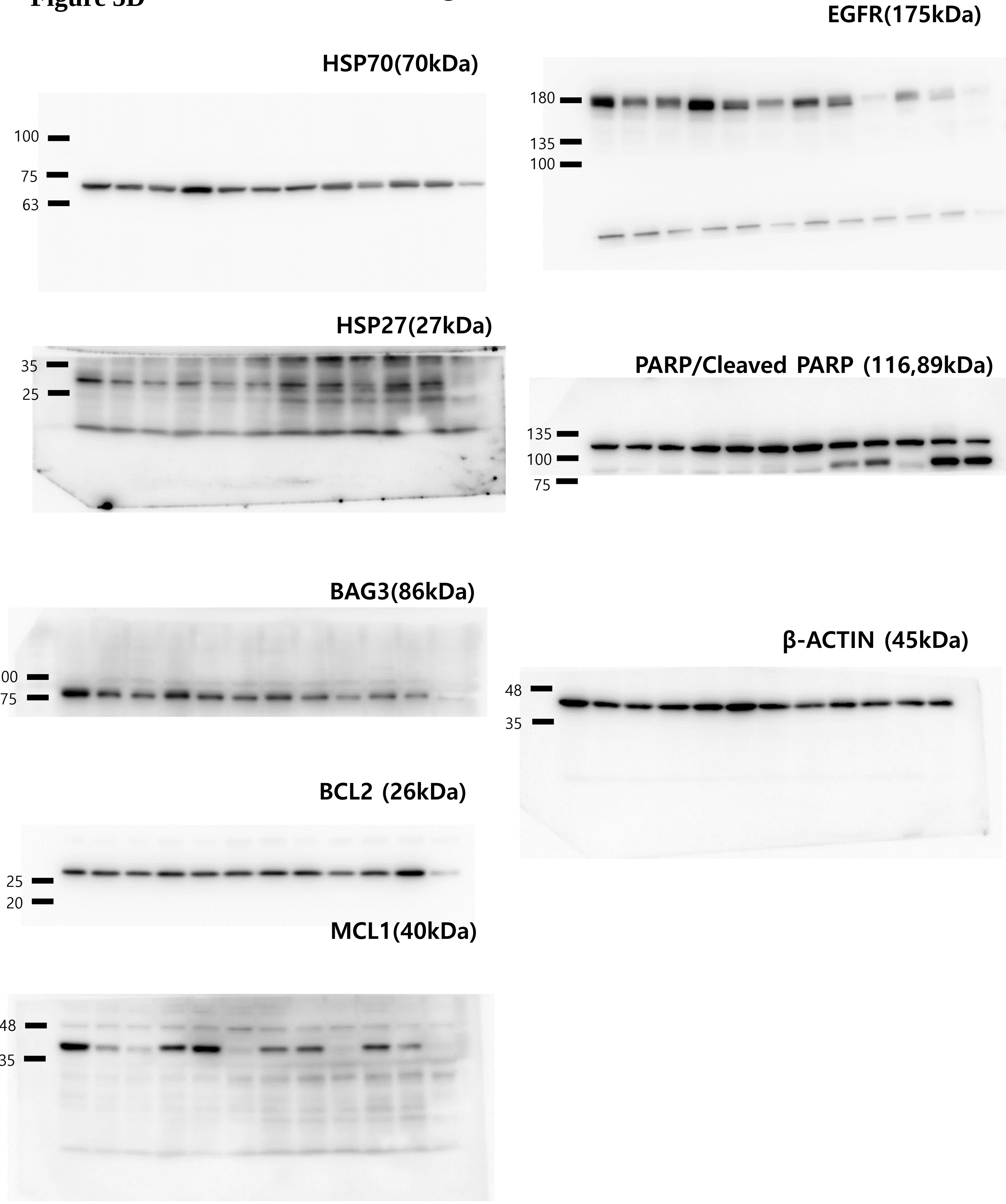
EGFR(175kDa)
180
135
100
HSP70(70kDa)
100
75
63
HSP27(27kDa)
35
25
PARP/Cleaved PARP (116,89kDa)
135
100
75
BAG3(86kDa)
00
75
β-ACTIN (45kDa)
48
35
BCL2 (26kDa)
25
20
MCL1(40kDa)
48
35

Figure 5D

Name	HSP70	HSP27	ACTIN	HSP70/ACT	HSP27/ACT
PC9-ErlR_0μM_12hr	30109.52	21392.77	45073.09	1.000000	1.000000
PC9-ErlR_10μM_12hr	24494.55	12110.73	35146.49	1.043280	0.726003
PC9-ErlR_20μM_12hr	24126.12	8867.14	32397.34	1.114786	0.576666
PC9-ErlR_0μM_24hr	42996.5	13325.92	38711.91	1.000000	1.000000
PC9-ErlR_10μM_24hr	25971.15	10323.45	43870.77	0.533000	0.683592
PC9-ErlR_20μM_24hr	23778.04	11454.27	49326.76	0.434015	0.674578
PC9-ErlR_0μM_48hr	25981.78	22220.07	38190.5	1.000000	1.000000
PC9-ErlR_10μM_48hr	26430.95	20894.12	27615.99	1.406820	1.300389
PC9-ErlR_20μM_48hr	16522.86	14846.63	34066.68	0.712922	0.749045
PC9-ErlR_0μM_72hr	24434.22	23408.74	29873.93	1.000000	1.000000
PC9-ErlR_20μM_72hr	21349.32	21610.85	28378.94	0.919775	0.971829
PC9-ErlR_20μM_72hr	7242.37	2819.08	27601.88	0.320801	0.130342

Name	BCL2	MCL1	ACTIN	BCL/ACT	MCL/ACT
PC9-ErlR_0μM_12hr	27326.78	40405.7	45073.09	1.000000	1.000000
PC9-ErlR_10μM_12hr	28449.62	8557.6	35146.49	1.335129	0.271609
PC9-ErlR_20μM_12hr	24172.81	3996.24	32397.34	1.230684	0.137600
PC9-ErlR_0μM_24hr	30987.13	26745.94	38711.91	1.000000	1.000000
PC9-ErlR_10μM_24hr	27776.58	41190.32	43870.77	0.790982	1.358960
PC9-ErlR_20μM_24hr	28402.57	9652	49326.76	0.719347	0.283218
PC9-ErlR_0μM_48hr	31350.64	21775.96	38190.5	1.000000	1.000000
PC9-ErlR_10μM_48hr	30414.9	25019.15	27615.99	1.341636	1.588876
PC9-ErlR_20μM_48hr	20622.73	8799.67	34066.68	0.737438	0.453017
PC9-ErlR_0μM_72hr	28425.01	25455.2	29873.93	1.000000	1.000000
PC9-ErlR_20μM_72hr	41814.13	13913.9	28378.94	1.548526	0.575398
PC9-ErlR_20μM_72hr	7329.26	4355.3	27601.88	0.279070	0.185181

Figure 5D

Name	BAG3	EGFR	ACTIN	BAG3/ACT	EGFR/ACT
PC9-ErlR_0μM_12hr	137.9	28519.63	45073.09	1.000000	1.000000
PC9-ErlR_10μM_12hr	77.23	17479.4	35146.49	0.718219	0.785992
PC9-ErlR_20μM_12hr	55.04	19179.08	32397.34	0.555293	0.935604
PC9-ErlR_0μM_24hr	94.4	36524.55	38711.91	1.000000	1.000000
PC9-ErlR_10μM_24hr	68.14	20000.67	43870.77	0.636941	0.483202
PC9-ErlR_20μM_24hr	54.14	12110.27	49326.76	0.450099	0.260214
PC9-ErlR_0μM_48hr	68.31	20349.98	38190.5	1.000000	1.000000
PC9-ErlR_10μM_48hr	51.5	16070.82	27615.99	1.042600	1.092116
PC9-ErlR_20μM_48hr	32.18	2138.27	34066.68	0.528114	0.117794
PC9-ErlR_0μM_72hr	53.96	9485.94	29873.93	1.000000	1.000000
PC9-ErlR_20μM_72hr	32.3	4557.47	28378.94	0.630125	0.505754
PC9-ErlR_20μM_72hr	0.25	863.01	27601.88	0.005014	0.098467

Figure 6C Sangah Lee, et al

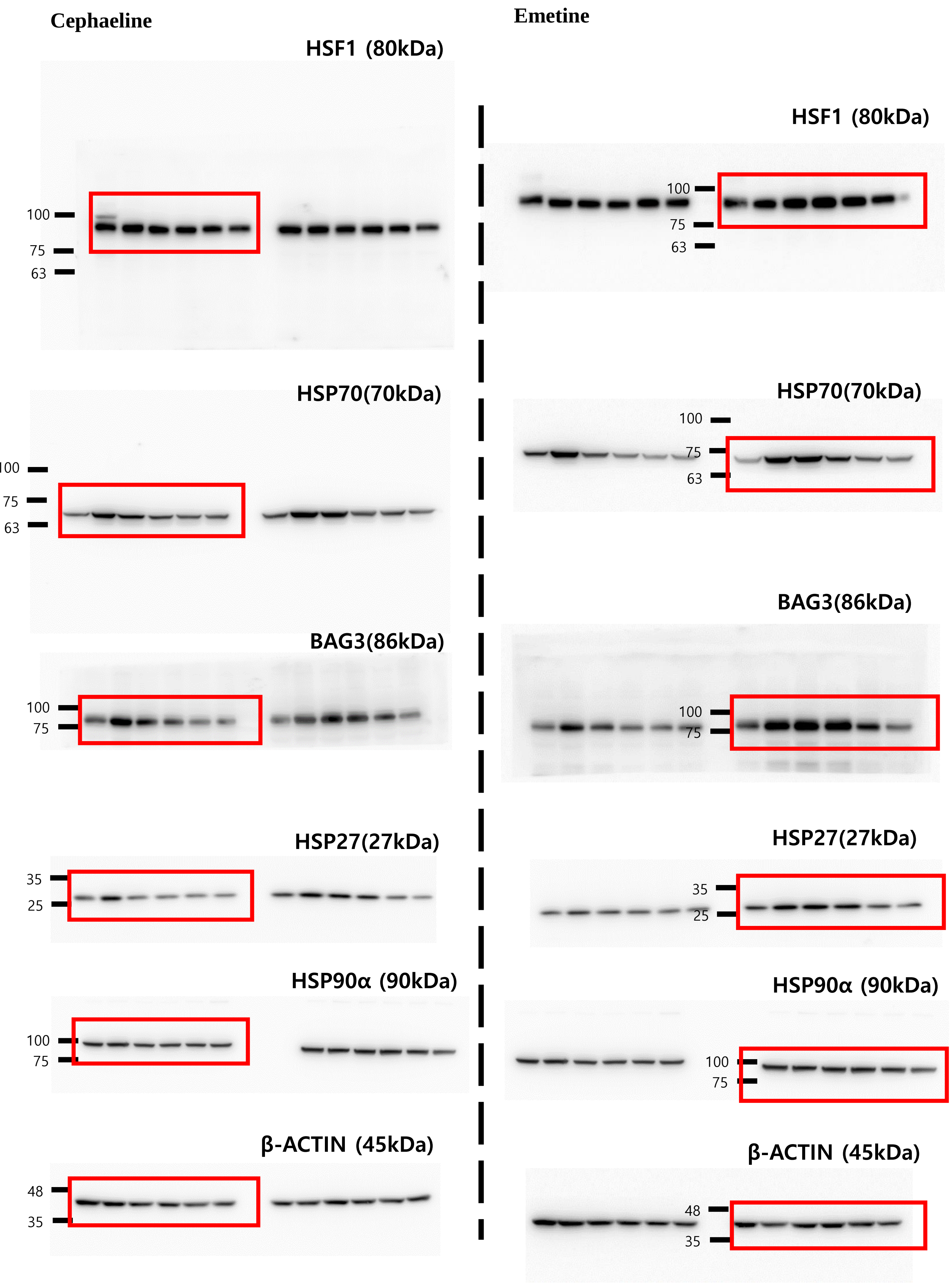

Cephaeline

Name	HSF1	HSP70	BAG3	ACTIN	HSF/ACT	H70/ACT	BAG/ACT
No-Heat 0nM	45312.72	15251.28	25889.14	34327.93	1.000000	1.000000	1.000000
Heat 0nM	49778.75	33824.83	46747.4	31731.64	1.188445	2.399299	1.953417
Heat 20nM	45554.38	30446.17	33952.07	24761.06	1.393761	2.767609	1.818139
Heat 50nM	43588.73	20784.52	25032.94	24448.85	1.350651	1.913475	1.357636
Heat 200nM	39126.05	18461.65	18209.56	21526.71	1.376943	1.930342	1.121636
Heat 500nM	34856.34	17920.75	15920.77	20966.45	1.259460	1.923856	1.006860

Name	HSP27	HSP90α	ACTIN	HSP27/ACT	HSP90α/ACT
No-Heat 0nM	17874.21	28077.88	34327.93	1.000000	1.000000
Heat 0nM	27351.82	31078.29	31731.64	1.655444	1.197424
Heat 20nM	14990.48	25280.23	24761.06	1.162698	1.248231
Heat 50nM	14120.88	25385.75	24448.85	1.109236	1.269448
Heat 200nM	13215.38	24520.69	21526.71	1.179024	1.392638
Heat 500nM	12472.49	25177.3	20966.45	1.142481	1.468140

Figure 6C Sangah Lee, et al

Emetine

Name	HSF1	HSP70	BAG3	ACTIN	HSF/ACT	H70/ACT	BAG/ACT
No-Heat 0nM	35726.3	11346.69	25567.51	32381.6	1.000000	1.000000	1.000000
Heat 0nM	41072.8	41187.69	44350.31	26628.04	1.398059	4.414254	2.109441
Heat 20nM	48714.53	43469.87	46191.87	31845.18	1.386517	3.895595	1.837095
Heat 50nM	51569.43	31246.25	45824.96	33941.92	1.377102	2.627185	1.709919
Heat 200nM	47225.03	21456.13	30959.77	31768.78	1.347355	1.927436	1.234261
Heat 500nM	37667.4	15646.02	18374.98	23558.57	1.449196	1.895327	0.987843

Name	HSP27	HSP90α	ACTIN	HSP27/ACT	HSP90α/ACT
No-Heat 0nM	26043.45	31602.25	32381.6	1.000000	1.000000
Heat 0nM	39318	32834.95	26628.04	1.835913	1.263506
Heat 20nM	41843.25	32586.53	31845.18	1.633735	1.048515
Heat 50nM	37726.85	33722.06	33941.92	1.382019	1.018024
Heat 200nM	23598.67	29963.55	31768.78	0.923606	0.966436
Heat 500nM	19097.58	23010.84	23558.57	1.007927	1.000838

Figure 6D

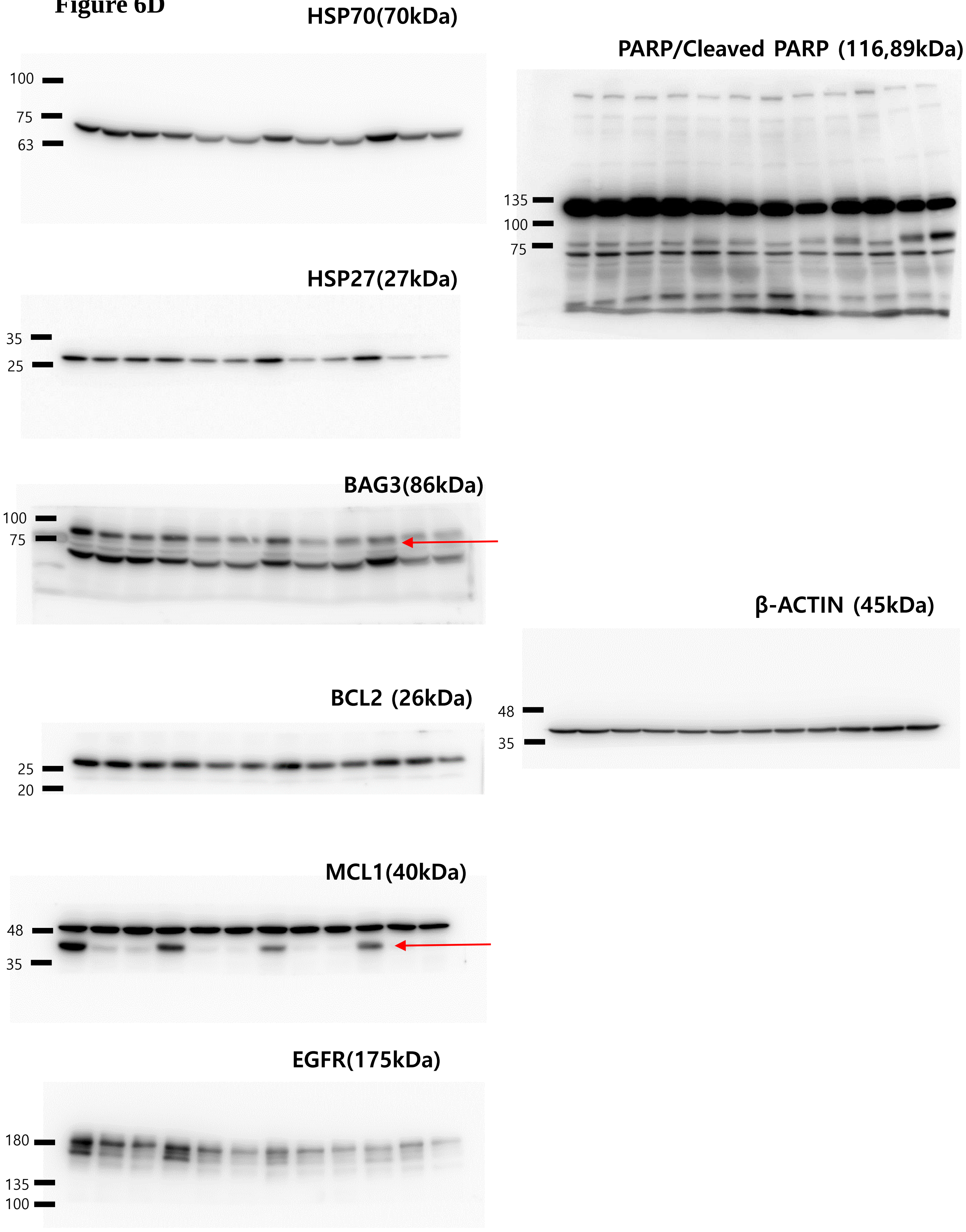
HSP70(70kDa)
100
75
63
PARP/Cleaved PARP (116,89kDa)
135
100
75
HSP27(27kDa)
35
25
BAG3(86kDa)
100
75
β-ACTIN (45kDa)
48
35
BCL2 (26kDa)
25
20
MCL1(40kDa)
48
35
EGFR(175kDa)
180
135
100

Figure 6D

Name	HSP70	HSP27	ACTIN	HSP70/ACT	HSP27/ACT
PC9-ErlR_0μM_12hr	27433.17	33414.42	18847.07	1.000000	1.000000
PC9-ErlR_0.5μM_12hr	33288.11	27378.04	20665.19	1.106669	0.747262
PC9-ErlR_2μM_12hr	30893.45	28482.05	17384.09	1.220906	0.924122
PC9-ErlR_0μM_24hr	25540.41	29321.84	16683.18	1.000000	1.000000
PC9-ErlR_0.5μM_24hr	18122.42	19362.74	15614.62	0.758116	0.705542
PC9-ErlR_1μM_24hr	18098.39	18108.37	15340.96	0.770617	0.671606
PC9-ErlR_0μM_48hr	27937.02	35585.81	16233.7	1.000000	1.000000
PC9-ErlR_0.5μM_48hr	20499.72	12414.73	17823.34	0.668338	0.317752
PC9-ErlR_1μM_48hr	20624.94	15437.87	18536.07	0.646565	0.379936
PC9-ErlR_0μM_72hr	37722.14	33189.11	23605.71	1.000000	1.000000
PC9-ErlR_0.5μM_72hr	23580.15	10980.66	26657.77	0.553533	0.292972
PC9-ErlR_1μM_72hr	20882.69	6930.92	25660.54	0.509262	0.192108

Name	BAG3	BCL2	ACTIN	BAG3/ACT	BCL2/ACT
PC9-ErlR_0μM_12hr	28870.97	39400.33	18847.07	1.000000	1.000000
PC9-ErlR_0.5μM_12hr	19773.39	39853.37	20665.19	0.624632	0.922507
PC9-ErlR_2μM_12hr	17963.01	34651.21	17384.09	0.674543	0.953477
PC9-ErlR_0μM_24hr	20268.02	33756.92	16683.18	1.000000	1.000000
PC9-ErlR_0.5μM_24hr	12683.13	24260.85	15614.62	0.668594	0.767875
PC9-ErlR_1μM_24hr	12665.3	25061.31	15340.96	0.679564	0.807360
PC9-ErlR_0μM_48hr	19634.59	36646.93	16233.7	1.000000	1.000000
PC9-ErlR_0.5μM_48hr	10125.08	28053.8	17823.34	0.469683	0.697240
PC9-ErlR_1μM_48hr	13503.12	24860.16	18536.07	0.602299	0.594109
PC9-ErlR_0μM_72hr	16358.11	34907.21	23605.71	1.000000	1.000000
PC9-ErlR_0.5μM_72hr	10373.17	30914.53	26657.77	0.561528	0.784225
PC9-ErlR_1μM_72hr	8600.97	16840.23	25660.54	0.483688	0.443797

Figure 6D

Name	MCL1	EGFR	ACTIN	MCL1/ACT	EGFR/ACT
PC9-ErlR_0μM_12hr	46095.58	41350.05	18847.07	1.000000	1.000000
PC9-ErlR_0.5μM_12hr	13224.32	35517.45	20665.19	0.261649	0.783376
PC9-ErlR_2μM_12hr	10607.58	29162.45	17384.09	0.249488	0.764610
PC9-ErlR_0μM_24hr	34270.73	38552.74	16683.18	1.000000	1.000000
PC9-ErlR_0.5μM_24hr	6366.16	25515.07	15614.62	0.198473	0.707113
PC9-ErlR_1μM_24hr	5462.44	16512.8	15340.96	0.173336	0.465792
PC9-ErlR_0μM_48hr	21529.23	22233.74	16233.7	1.000000	1.000000
PC9-ErlR_0.5μM_48hr	5388.4	17238.61	17823.34	0.227961	0.706184
PC9-ErlR_1μM_48hr	5520.73	12763.87	18536.07	0.224578	0.502770
PC9-ErlR_0μM_72hr	24492.51	12521.86	23605.71	1.000000	1.000000
PC9-ErlR_0.5μM_72hr	6373.2	14773.49	26657.77	0.230419	1.044738
PC9-ErlR_1μM_72hr	4446.24	6837.3	25660.54	0.166998	0.502304

Figure 6G

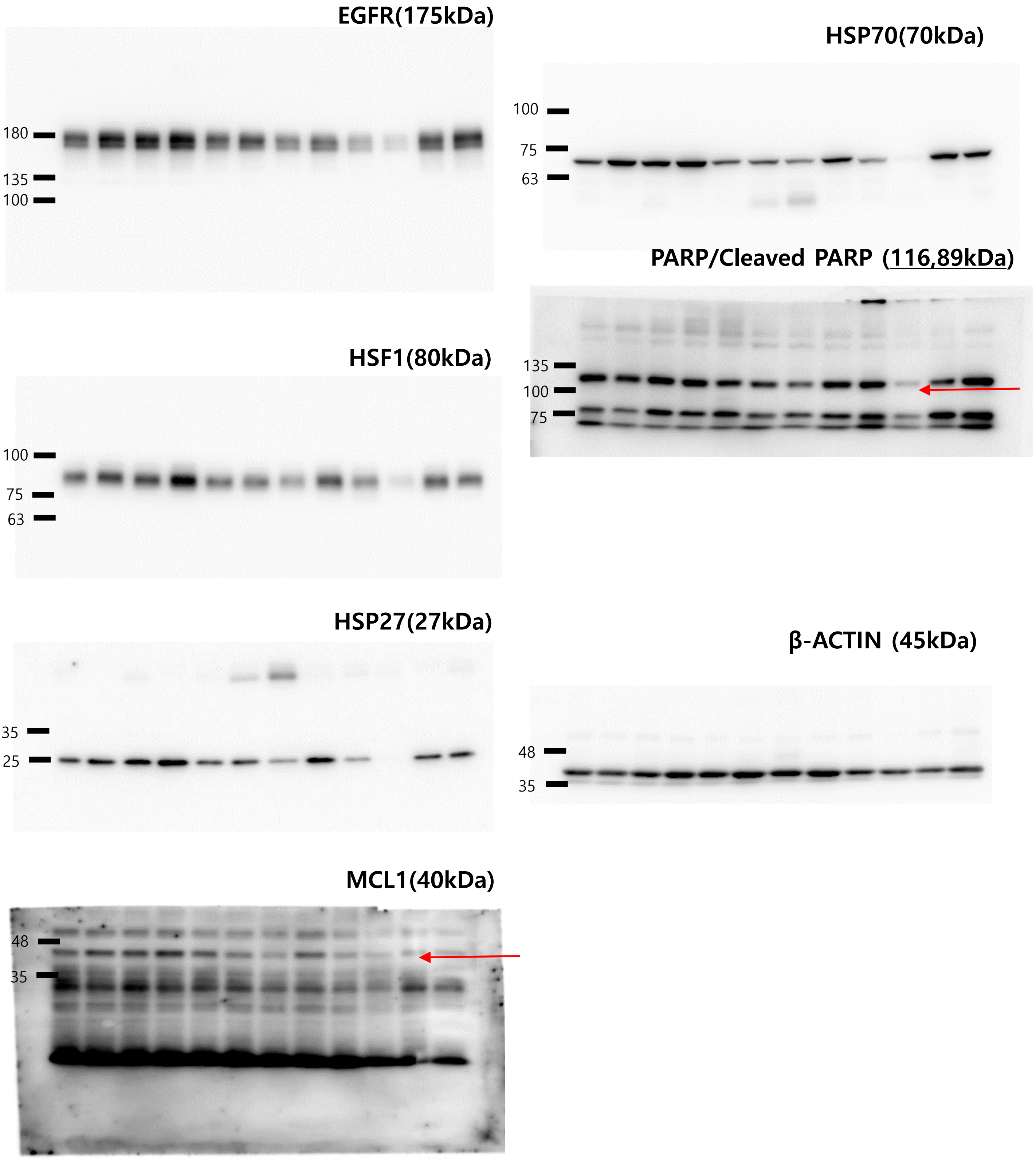
EGFR(175kDa)
180
135
100
HSP70(70kDa)
100
75
63
PARP/Cleaved PARP (116,89kDa)
135
100
75
HSF1(80kDa)
100
75
63
HSP27(27kDa)
35
25
β-ACTIN (45kDa)
48
35
MCL1(40kDa)
48
35

Figure 6G

Name	EGFR	HSF1	ACTIN	EGFR/ACT	HSF1/ACT
Vehicle #`1	90.74	77.29	97.66	1.000000	1.000000
Vehicle #`2	130.22	106.71	100.68	1.392042	1.339230
Vehicle #`3	124.93	103.85	122.27	1.099676	1.073198
Vehicle #`4	139.91	151.87	146.77	1.025958	1.307459
Vehicle #`5	87.08	79.68	139.28	0.672895	0.722860
Vehicle #`6	96.9	77.8	156.8	0.665113	0.626941
Emetine #1	63.84	50.7	136.39	0.503765	0.469698
Emetine #2	81.46	101.52	150.92	0.580919	0.849959
Emetine #3	41.45	62.07	100.78	0.442658	0.778217
Emetine #4	20.78	18.46	92.25	0.242436	0.252848
Emetine #5	95.29	90.47	83.61	1.226612	1.367224
Emetine #6	125.07	81.54	112.72	1.194181	0.914036

Name	HSP27	MCL1	ACTIN	HSP27/ACT	MCL1/ACT
Vehicle #`1	73.45	43.54	97.66	1.000000	1.000000
Vehicle #`2	111.17	63.69	100.68	1.468146	1.418915
Vehicle #`3	123.84	59.78	122.27	1.346685	1.096641
Vehicle #`4	154.1	78.17	146.77	1.396016	1.194624
Vehicle #`5	75.14	52.05	139.28	0.717311	0.838224
Vehicle #`6	85.56	38.6	156.8	0.725520	0.552166
Emetine #1	41.34	16.15	136.39	0.403007	0.265594
Emetine #2	124.31	47.94	150.92	1.095177	0.712491
Emetine #3	36.17	23.14	100.78	0.477199	0.515012
Emetine #4	2.77	21.85	92.25	0.039924	0.531268
Emetine #5	79.4	23.63	83.61	1.262662	0.633919
Emetine #6	84.28	27.12	112.72	0.994142	0.539656

Figure 6G

Name	HSP70	PARP	ACTIN	HSP70/ACT	PARP/ACT
Vehicle #`1	23534.61	114.55	97.66	1.000000	1.000000
Vehicle #`2	33361.04	93.99	100.68	1.375011	0.795903
Vehicle #`3	33818.7	117.04	122.27	1.147748	0.816086
Vehicle #`4	35420.39	110.69	146.77	1.001442	0.642973
Vehicle #`5	18012.94	94.32	139.28	0.536668	0.577347
Vehicle #`6	19834.86	84.72	156.8	0.524920	0.460640
Emetine #1	13917	59.99	136.39	0.423421	0.374989
Emetine #2	27196.87	115.34	150.92	0.747794	0.651561
Emetine #3	12055.86	112.17	100.78	0.496402	0.948908
Emetine #4	1712.84	22.45	92.25	0.077048	0.207478
Emetine #5	29733.06	66.84	83.61	1.475676	0.681553
Emetine #6	28023.86	139.12	112.72	1.031660	1.052229
