# Supplementary material for: Targeting HSF1 as a Therapeutic Strategy for Multiple Mechanisms of EGFR Inhibitor Resistance in EGFR Mutant Non-Small-Cell Lung Cancer
Source: Cancers (Basel). 2021 Jun 15;13(12):2987. doi: 10.3390/cancers13122987 (PMC8232331; doi:10.3390/cancers13122987)

Supplementary Materials:

Table S1: List of 4-fold up-regulated genes in HCC827-ErlR cells compared with HCC827 parental cells.
-Exel file

Table S2: List of 6.5-fold down-regulated genes in HCC827-ErlR cells compared with HCC827 parental cells.
-Exel file

Figure S1: The sequencing of PC9-ErlR and HCC827-ErlR on the EGFR (T790) region

Figure S2: The whole blot showing all the bands with molecular weight marker.

Figure S1: The sequencing of PC9-ErlR and HCC827-ErlR on the EGFR (T790) region

A. PC9-ErlR cDNA sequencing

PC9-ElrR cells were plated in 96-well plates at a density of 0.5 cells per well. After a single colony was formed in each well, we selected and grew 10 subclones from PC9-ErlR cells. For sequencing, each subclone was plated in a 60 mm dish at a density of 4 x 10^5 cells per well. After 24 h, total RNA was isolated using the RNeasy kit (Qiagen). For each subclone, 1 microgram of isolated RNA was reverse transcribed with the RevertAid First Strand cDNA Synthesis Kit (Toyobo, Osaka, Japan) according to the manufacturer's instructions. Thereafter, the EGFR exon 20 region was amplified by PCR using EGFR-specific primers, and the PCR product was sequenced by Solgent Co. (Daejeon, Republic of Korea). The following primers were used for PCR amplification: EGFR exon 20 forward primer, 5'-CCCAACCAAGCTCTCTTGAG-3' and EGFR exon 20 reverse primer, 5'-ATGACAAGGTAGCGCTGGGG-3'.

Supplementary Figure 1.

A. PC9-ErlR cDNA sequencing data

Single cell isolation---total RNA isolation---cDNA synthesis---
PCR amplification for EGFR Exon 20---PCR product was sequenced.
(for detail, see Materials and Methods)

```
               781      790          800
               |--------+---------+-
   TotalEGFR   CLTSTVQLITQLMPFGCLLDY
  EGFRexon20   CLTSTVQLITQLMPFGCLLDY
  PC9-ElrR(8)  CLTSTVQLIXQLMPFGCLLDY
  PC9-ElrR(1)  CLTSTVQLIXQLMPFGCLLDY
  PC9-ElrR(3)  CLTSTVQLIXQLMPFGCLLDY
  PC9-ElrR(7)  CLTSTVQLIXQLMPFGCLLDY
  PC9-ElrR(9)  CLTSTVQLIXQLMPFGCLLDY
  PC9-ElrR(4)  CLTSTVQLIXQLMPFGCLLDY
  PC9-ElrR(6)  CLTSTVQLIXQLMPFGCLLDY
  PC9-ElrR(5)  CLTSTVQLIXQLMPFGCLLDY
  PC9-ElrR(2)  CLTSTVQLIXQLMPFGCLLDY
 PC9-ElrR(10)  CLTSTVQLIXQLMPFGCLLDY
   Consensus   CLTSTVQLIXQLMPFGCLLDY
```

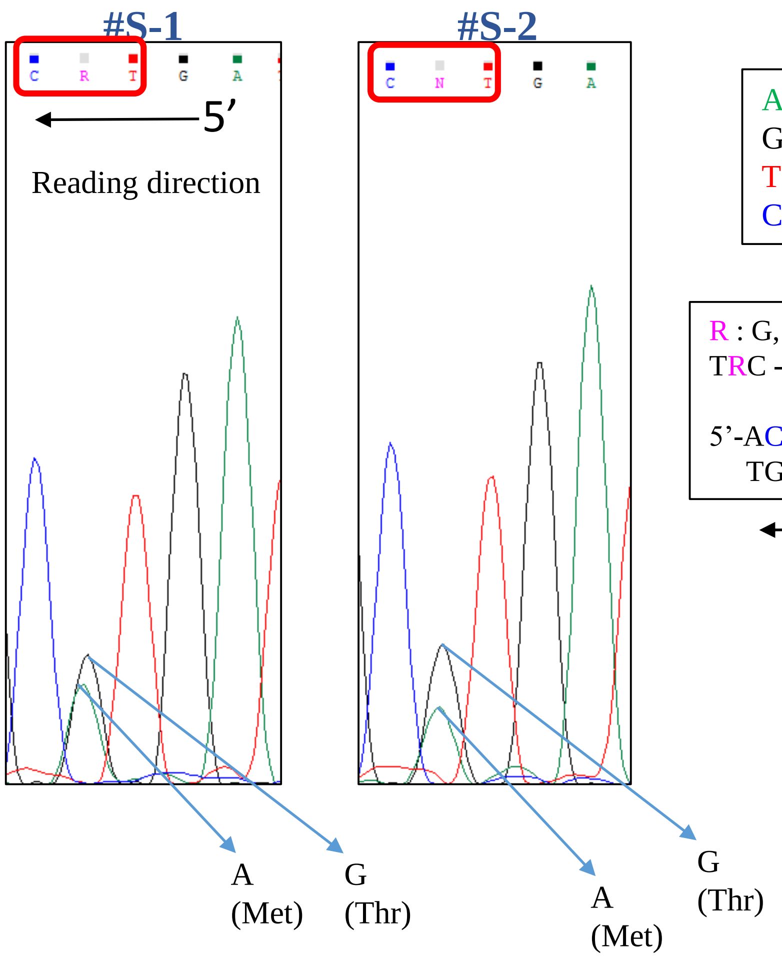

A : green
G : black
T : red
C : blue

R : G, A -> C, T
TRC ->

5’-ACG (Thr), TGC-5’

5’-ATG(Met) TAC-5’

5’ Reading direction

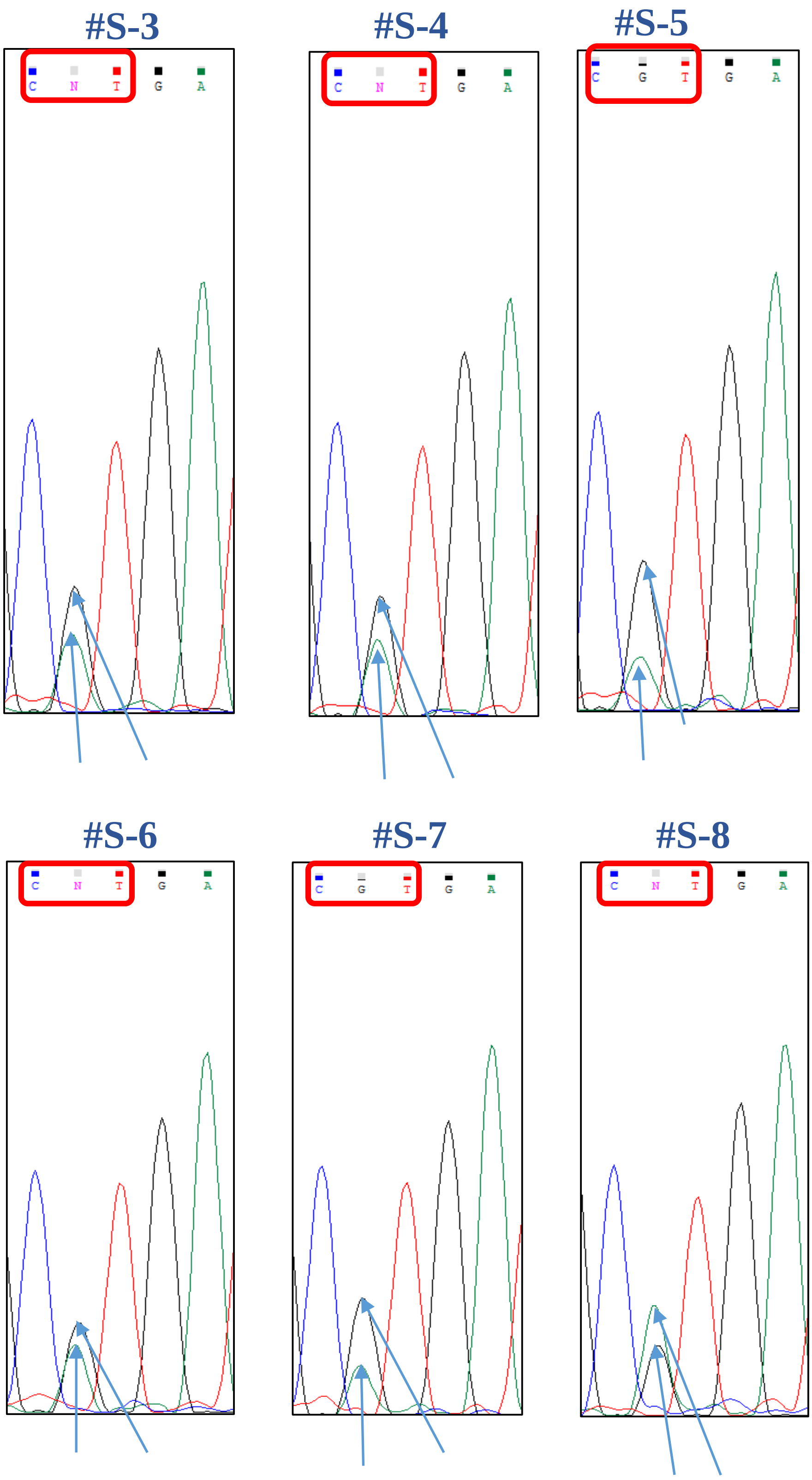
#S-3
C N T G A
#S-4
C N T G A
#S-5
C G T G A
#S-6
C N T G A
#S-7
C G T G A
#S-8
C N T G A

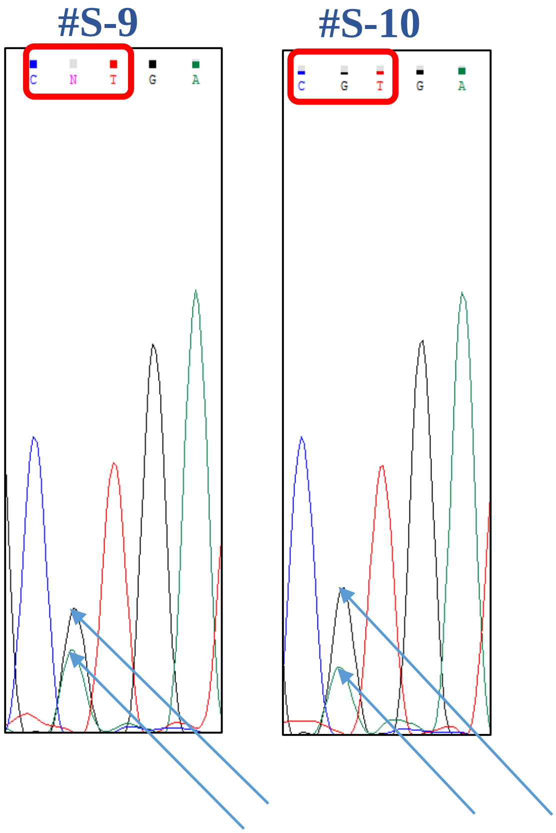
#S-9
C N T G A
#S-10
C G T G A

B. HCC827-ErlR cDNA sequencing data

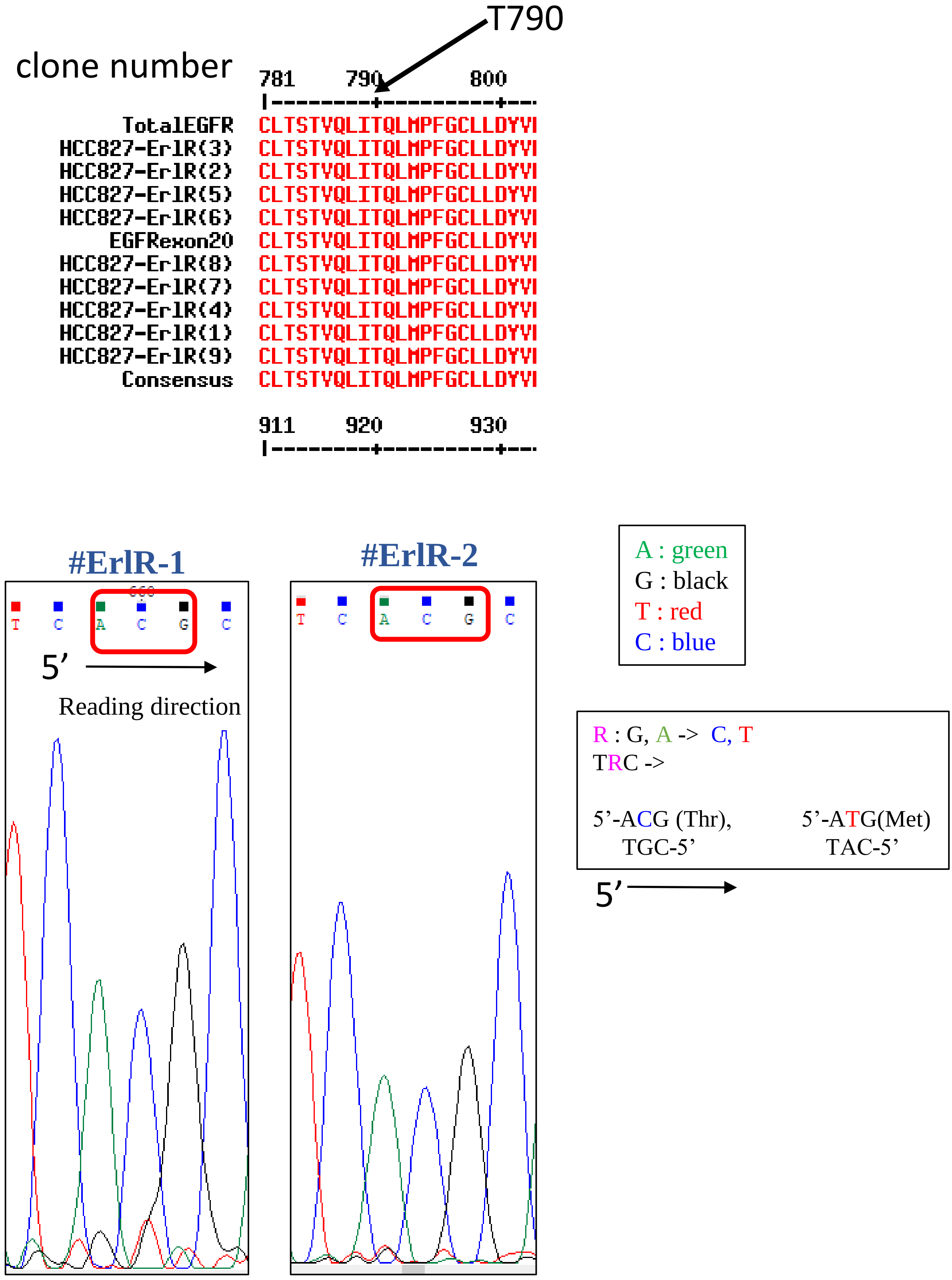

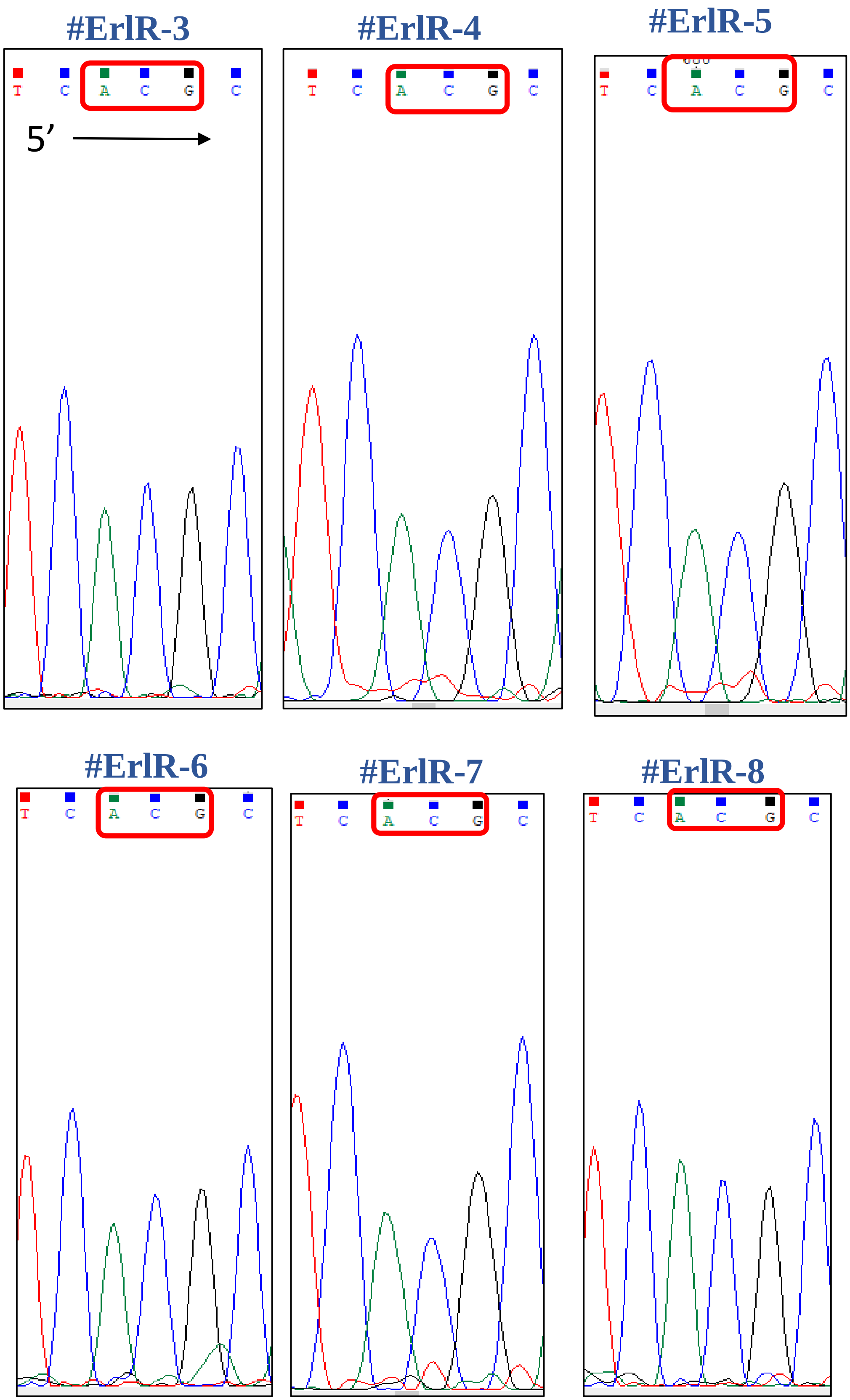

#ErlR-3
T C A C G C
5’
#ErlR-4
T C A C G C
#ErlR-5
T C A C G C
#ErlR-6
T C A C G C
#ErlR-7
T C A C G C
#ErlR-8
T C A C G C

#ErlR-9

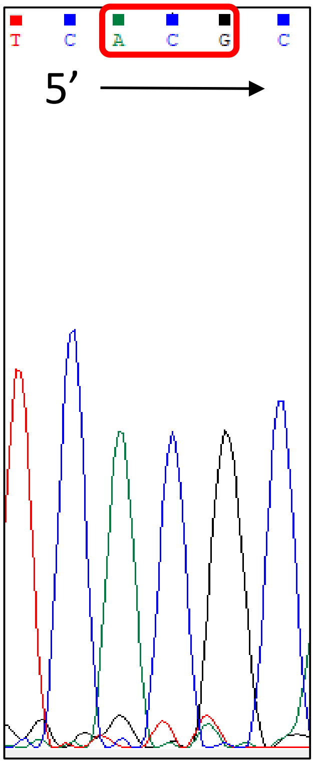

Supplement: Supplementary file 1 [file cancers-13-02987-s001.zip › cancers-1253451-supplementary/Figure S1 Sequencing for Lee et al .pdf]
